# The sensitivity of real-time PCR amplification targeting invasive *Salmonella *serovars in biological specimens

**DOI:** 10.1186/1471-2334-10-125

**Published:** 2010-05-21

**Authors:** Tran Vu Thieu Nga, Abhilasha Karkey, Sabina Dongol, Hang Nguyen Thuy, Sarah Dunstan, Kathryn Holt, Le Thi Phuong Tu, James I Campbell, Tran Thuy Chau, Nguyen Van Vinh Chau, Amit Arjyal, Samir Koirala, Buddha Basnyat, Christiane Dolecek, Jeremy Farrar, Stephen Baker

**Affiliations:** 1 Oxford University Clinical Research Unit, Hospital for Tropical Diseases, Ho Chi Minh City, Vietnam; 2 The Hospital for Tropical Diseases, Ho Chi Minh City, Vietnam; 3 Oxford University Clinical Research Unit, Patan Academy of Health Sciences, Kathmandu, Nepal; 4 Wellcome Trust Major Overseas Programme, Ho Chi Minh City, Vietnam; 5 Department of Microbiology and Immunology, The University of Melbourne, Victoria, Australia

## Abstract

**Background:**

PCR amplification for the detection of pathogens in biological material is generally considered a rapid and informative diagnostic technique. Invasive *Salmonella *serovars, which cause enteric fever, can be commonly cultured from the blood of infected patients. Yet, the isolation of invasive *Salmonella *serovars from blood is protracted and potentially insensitive.

**Methods:**

We developed and optimised a novel multiplex three colour real-time PCR assay to detect specific target sequences in the genomes of *Salmonella *serovars Typhi and Paratyphi A. We performed the assay on DNA extracted from blood and bone marrow samples from culture positive and negative enteric fever patients.

**Results:**

The assay was validated and demonstrated a high level of specificity and reproducibility under experimental conditions. All bone marrow samples tested positive for *Salmonella*, however, the sensitivity on blood samples was limited. The assay demonstrated an overall specificity of 100% (75/75) and sensitivity of 53.9% (69/128) on all biological samples. We then tested the PCR detection limit by performing bacterial counts after inoculation into blood culture bottles.

**Conclusions:**

Our findings corroborate previous clinical findings, whereby the bacterial load of *S. *Typhi in peripheral blood is low, often below detection by culture and, consequently, below detection by PCR. Whilst the assay may be utilised for environmental sampling or on differing biological samples, our data suggest that PCR performed directly on blood samples may be an unsuitable methodology and a potentially unachievable target for the routine diagnosis of enteric fever.

## Background

The detection of invasive *Salmonella *serovars such as *Salmonella *Typhi (*S. *Typhi) and *Salmonella *Paratyphi A (*S*. Paratyphi A) remains a challenging problem. Depending on the location, various different tests and clinical criteria are used to distinguish febrile disease of differing aetiology, many of which still may remain unsatisfactorily identified. In resource poor settings with a high disease burden, enteric fever is largely distinguished on the basis of clinical symptoms and syndromes [1-4]. Yet, clinical symptoms are not the most reliable assessment for enteric fever, as other conditions, such as typhus, malaria and leptospirosis have similar clinical manifestations and are also common in places such as Nepal [5,6].

The current WHO guidelines for typhoid fever states that "The definitive diagnosis of typhoid fever depends on the isolation of *S*. Typhi from blood, bone marrow or a specific anatomical lesion" and concludes "Blood culture is the mainstay of the diagnosis of this disease" [7]. However, in practice, neither blood or bone marrow culture is performed routinely. Many hospitals in resource limited settings do not have adequate microbiological laboratory facilities and personnel to perform such a technique. Our current unpublished data suggests that only 40% of patients with a clinical syndrome indicative of enteric fever attending Patan Hospital in Kathmandu are culture positive for invasive *Salmonellae*. The culturing of bone marrow biopsies from enteric fever patients has a higher sensitivity than blood culture (between 70% and 80% on clinically diagnosed cases [8,9]) but is seldom performed due to the aggressive nature of the investigation. Culturing biological specimens from patients can also not be considered rapid; it may take between one and three days for positive blood culture and a further one to two days for identification and antimicrobial resistance profiling.

We aimed to develop, initially for research purposes, a robust and rapid test for the identification of *S. *Typhi and *S. *Paratyphi A in biological specimens, with the possibility that it may form the basis of a suitable diagnostic test in the future. PCR offers a potentially attractive methodology for the detection of invasive *Salmonella *serovars. PCR amplification is commonly used in many clinical research laboratories for the detection of multiple pathogens. Furthermore, there are several publications demonstrating the utility of PCR for the detection of invasive *Salmonella *serovars in the blood [10-14]. Yet, the use of PCR for the definitive diagnosis of enteric fever is somewhat contentious, despite the method being previously referred to as "the gold standard for diagnosis" [15]. Our understanding is that PCR is not routinely performed in areas with endemic enteric fever and the invalidated methodology means PCR should be not considered a reliable method for diagnosis or for measuring disease burden.

Here we address some of the issues with the use of PCR for detection of invasive *Salmonella *serovars, and consider if this methodology could evolve into a standardised test that may be used as a complementary diagnostic tool in the future. Therefore, we developed a novel multiplex real-time PCR assay that would amplify specific DNA sequences from *S*. Typhi and *S*. Paratyphi A. We then tested the methodology on biological samples collected from enteric fever patients.

## Methods

### Patient selection, blood and bone marrow sampling

Blood samples were collected from patients presenting to Patan Hospital, Kathmandu, Nepal with suspected uncomplicated enteric fever that had not taken antimicrobials prior to admission. Bone marrow specimens were taken from patients admitted to the Hospital for tropical diseases in Ho Chi Minh City, Vietnam with suspected enteric fever. The study was approved by the scientific committees and ethical committees of the participating institutions. Written informed consent was obtained from all participants or guardians of participants. Samples of 10 ml of anti-coagulant blood were collected in EDTA tubes from febrile patients over the age of 12 years old; 6 ml was used for the isolation of *Salmonella *serovars by routine blood culture. The remaining 4 ml was centrifuged at 1,100 RCF for 10 minutes and the plasma and whole blood cell pellets were separated and stored at -80°C. Bone marrow biopsies were taken as previously described [16], bone marrow was cultured for the isolation of *Salmonella *serovars and 1 ml of tissue was stored at -80°C until DNA extraction.

### Target sequence selection

Sequences unique to *S. *Typhi or *S. *Paratyphi A were identified using a whole-genome comparison of *S. *Typhi strain CT18 (GenBank AL513382) [17] and *S. *Paratyphi A strain AKU12601 (GenBank FM200053) [18], conducted using BLASTn and visualized using the Artemis Comparison Tool (ACT). To confirm whether these sequences were likely to discriminate more generally between members of the *S. *Typhi and *S. *Paratyphi A populations, we searched for sequences in all available *S. *Typhi (finished sequence for strain Ty2 (GenBank AE014613) and 17 additional 454 shotgun-sequenced strains (GenBank CAAV01000001-CAAV01003682)) [19] and *S. *Paratyphi A strains (finished sequence for strain ATCC9150 (GenBank CP000026)). Genomic data from the recent *S. *Typhi and *S. *Paratyphi A sequencing projects were mined to find genes that were specific for each serovar [18,19]. The criteria for selection were; a lack of homology with other genes in other pathogens or human sequences (to ensure no cross-reactivity) and the sequence was required to be conserved in all the re-sequenced and previously sequenced strains.

### DNA manipulation, bacterial strains and construction of internal control

All bacterial strains used in this study are presented in Table 1. Strain *E. coli *VU1 was constructed by PCR amplifying the gB gene from Phocid herpes virus using the primers phHV-1 forward and reverse [20]. The gB gene amplicon was cloned into plasmid pCR 2.1-TOPO (Invitrogen). *E. coli *VU1 was to act as an internal control to monitor DNA extraction and amplification efficiency in all PCR reactions using primers phHV-1 forward and reverse and a specific probe [20]. PCR amplicons for all target sequences were produced by monoplex conventional PCR using the primer sequences outlined below. *E. coli *TOP10 cells (Invitrogen) were transformed with purified plasmid DNA containing target DNA sequence and PCR amplicons were sequenced (Applied Biosystems) to ensure accurate amplification. Purified plasmid DNA was used as template in all subsequent experiments which utilized a standard curve.

**Table 1:** Bacterial strains used in this study

Strains	Number	Description/Source
**Laboratory isolates**		

*Salmonella *Typhi CT18	-	Sanger Institute collection
*Salmonella *Paratyphi A AKU12601	-	Sanger Institute collection
*E. coli *Vu 1	-	Cloned gB target sequence - this study
*E. coli *Vu 2	-	Cloned STY0201 target sequence - this study
*E. coli *Vu 3	-	Cloned SSPA2308 target sequence - this study

**Clinical isolates**		

*Salmonella *Typhi	80	OUCRU Nepal
*Salmonella *Paratyphi A	60	OUCRU Nepal
*Staphylococcus. spp*	3	OUCRU Vietnam
*Streptococcus pneumoniae*	1	OUCRU Vietnam
*Streptococcus suis *type 2	2	OUCRU Vietnam
*Streptococcus *group B	1	OUCRU Vietnam
*Neisseria meningitidis*	1	OUCRU Vietnam
*Citrobacter freundii*	4	OUCRU Vietnam
*Klebsiella pneumoniae*	1	OUCRU Vietnam
*Salmonella *serotypes	10	OUCRU Vietnam

### Total genomic and plasmid DNA extraction

Volumes of 200 μl to 2 ml of experimental blood samples (for laboratory assessment) were used for total DNA extraction. From patient samples, we consistently used 2 ml of blood cell pellets and 1 ml of bone marrow biopsies spiked with 50 μl of *E. coli *VU1 for total DNA isolation. Extractions were performed under sterile conditions using the QIAamp DNA Blood Midi Kit (Qiagen) according to the manufacturer's recommendations. DNA was re-suspended in 300 μl of elution buffer, stored at 4°C and subjected to PCR within 24 hours of preparation. Plasmid DNA was purified from *E. coli *VU1 and from strains containing PCR target DNA using the QIAprep Spin Miniprep (Qiagen) according to the manufacturer's recommendations. In total, the PCR assay was performed on blood samples from 100 patients with blood culture confirmed enteric fever, 50 blood samples from patients with presumptive enteric fever (blood culture negative), 25 patients with bacteraemia caused by organisms other than *S. *Typhi or *S. *Paratyphi A and 28 bone marrow biopsies from patients with culture confirmed enteric fever cause by *S*. Typhi.

### Primers and PCR conditions

Primers and probes specific A were designed using Primer Express Software (Applied biosystems) and manufactured by Sigma -Proligo (Singapore). Primers and probes sequences were as follows; *S. *Typhi; ST-Frt 5' CGCGAAGTCAGAGTCGACATAG 3', ST-Rrt 5' AAGACCTCAACGCCGATCAC 3', ST- Probe 5' FAM-CATTTGTTCTGGAGCAGGCTGACGG-TAMRA 3'; *S. *Paratyphi A; Pa-Frt 5'ACGATGATGACTGATTTATCGAAC 3', Pa-Rrt 5' TGAAAAGATATCTCTCAGAGCTGG 3', Pa-Probe 5' Cy5-CCCATACAATTTCATTCTTATTGAGAATGCGC-BHQ5 3' and Phocid herpes virus; PhHV-Frt 5' GGGCGAATCACAGATTGAATC 3', PhHV-Frt 5' GCGGTTCCAAACGTACCAA 3', phHV-Probe-hex 5' Hex-TTTTTATGTGTCCGCCACCATCTGGATC-TAMRA 3'.

PCR reactions were performed in 25 μl reaction volumes consisting of 5 mM MgCl_2_, 0.2 mM each deoxynucleotide triphosphate, 1 U of Hot start Taq DNA polymerase (Qiagen) and 5 μl of template DNA. Final reaction concentrations of the three primer and probe sets for internal control, *S. *Typhi and *S. *Paratyphi A were 0.4 μM of each primer and 0.15 μM of each probe. PCR was performed on a Bio-Rad Chromo 4 real-time PCR system and fluorescence was released via the TaqMan 5' to 3' exonuclease activity. All PCRs were cycled under the following conditions; 15 min at 95°C and 45 cycles of 30 sec at 95°C, 30 sec at 60°C and 30 sec at 72°C.

### Real-time PCR, quantification, reproducibility and interpretation

Plasmid DNA with cloned target DNA sequences (*S. *Typhi and *S. *Paratyphi A) were purified and concentrations (μg/ml) were calculated by a NanoDrop spectrophotometer (Thermo-Scientific). Concentrations were converted to copy number using the formula; mol/g × molecules/mol = molecules/g, via a DNA copy number calculator http://www.uri.edu/research/gsc/resources/cndna.html. Plasmid solutions were diluted in 10-fold serial dilutions ranging from 10^0 ^to 10^5 ^plasmid copies per μl. Serially diluted plasmid DNA was mixed in increasing (*S. *Typhi target) and decreasing (*S. *Paratyphi A target) concentrations and subjected to real-time PCR amplification. Standard curves for *S. *Typhi and *S. *Paratyphi A copy number were constructed by plotting the *Ct *value against the plasmid DNA copy number. The intra-assay co-efficient of variance was calculated by the assessing deviation in *Ct *values of a selected plasmid concentration. This was performed using four replicates all of which were amplified on the same day. Inter-assay variation was calculated by measuring the variation in *Ct *values of selected concentrations over a four day period. Accurate DNA extraction and amplification was confirmed in all experiments by production of a green signal from the internal control. A negative PCR result was concluded if negative controls were negative, the internal control showed an expected *Ct *value and the reporter signal for *S. *Typhi or *S. *Paratyphi A could not be detected. Data was deemed none-interpretable when the negative control demonstrated contamination and/or the internal control did not yield a sufficient *Ct *value. For each run of the real-time PCR assay, DNA from *S. *Typhi CT18 and *S. *Paratyphi A AKU12601 was included as a positive control in the assay plate. All statistical analyses were performed in R http://www.r-project.org/.

### Laboratory assay for detection limits

The experimental detection limit was calculated using two methodologies and subsequent results were compared to assess variability. Initially, 10 ml cultures of *S. *Typhi and *S. *Paratyphi A were grown overnight with aeration at 37°C in Luria-Bertani media and 200 μl was used to inoculate one ml of whole blood. Serial dilutions of the inoculated blood samples were made and the bacterial suspensions were concurrently serially diluted in phosphate buffered saline. 200 μl of each dilution of saline and the corresponding blood specimen was used for total DNA extraction (as described above). DNA was re-suspended in 200 μl of elution buffer. Bacterial counts for each bacterial dilution in blood and saline were performed in triplicate and enumerated on Luria-Bertani media. The numbers of colony forming units were compared to the *Ct *value following real-time PCR amplification. Additionally, real-time PCR was performed on serial dilutions of isolated plasmid DNA containing cloned target sequences until a positive signal could no longer be detected from the assay.

### Blood inoculation experiment

One ml of whole blood was inoculated with 200 μl of an overnight culture of *S. *Typhi (as above) and was equilibrated at 37°C with agitation for 16 hours. Serial dilutions of the inoculated blood sample were performed concurrently in phosphate buffered saline and whole blood. The resulting bacterial dilutions were enumerated on Luria-Bertani media and total DNA was extracted from 200 μl of diluted blood and PBS. Additionally, the remaining diluted blood samples were inoculated into 25 ml BACTEC Plus aerobic bottles (Becton - Dickinson) and incubated at 37°C in a BACTEC 9050 machine (Becton Dickinson) as per the manufacturers recommendations, until growth was detected. Any cultured organisms were sub-cultured to ensure no contamination. This experiment was also performed on inoculated whole blood individually treated with gentamycin and ciprofloxacin. Blood was inoculated as before and either gentamycin or ciprofloxacin (Sigma Aldrich) was added (to a final concentration of 100 μg/ml) and incubated at 37°C for two hours.

## Results

### Optimisation of a three color multiplex real-time PCR assay

We were able to identify several potential DNA sequence targets that were specific to *S. *Typhi or *S. *Paratyphi A and demonstrated no DNA homology to other sequences found in database searches. Ultimately, we selected an individual coding sequence target from each of the two serovars. These were; STY0201 from *S*. Typhi, (encoding a putative fimbrial-like adhesin protein located at position 210,264 in the *S. *Typhi CT18 chromosome genome sequence (Accession number NC_003198)) and SSPA2308 from *S*. Paratyphi A (encoding a hypothetical protein at position 2,572,177 in the *S. *Paratyphi A AKU_12601 chromosome (Accession number FM200053)).

The *S. *Typhi specific primers were predicted to produce a 131 bp amplicon from within gene STY0201 and the *S. *Paratyphi A primers were predicted to amplify a 104 bp fragment within the gene SSPA2308. PCR reactions were optimized and multiplexed. Strain *E. coli *incorporating the VU1 phocid virus gene was added to ensure accurate DNA extraction from all specimens and to act as a positive control during amplification. An *E. coli *or *Salmonella *gene target was deemed inappropriate due to obvious cross hybridization problems.

The serovar specific loci were present in all available genome sequences and we ensured the presence of the target sequences on DNA extracted from 140 *S. *Typhi and *S. *Paratyphi A strains by PCR (these strains included the 100 strains isolated from blood specimens used in later experiments). Prior to extraction, the bacterial cultures were spiked with 200 μl of *E. coli *VU1. To control for potential cross reactivity, 10 other *Salmonella *serovars (including Enteritidis, Typhimurium and Paratyphi C) and 13 other bacterial pathogens commonly isolated during blood culture, including *Staphylococcus aureus *and *Streptococcus pneumoniae *(Table 1) were tested for the presence of the DNA sequences.

When the real-time PCR amplification was performed on DNA prepared from either *S. *Typhi or *S. *Paratyphi A the assay demonstrated serovar specific amplification on all tested DNA samples. We could detect a positive internal control signal in all amplifications and were unable to detect amplification of the *S. *Typhi and *S. *Paratyphi A target sequences in DNA from other *Salmonella *serovars or other bacterial pathogens (data not shown). Therefore, on extracted DNA, the real-time PCR assay demonstrated good specificity. The final assay conditions demonstrated no cross-hybridization when performed individually on DNA extracted from *E. coli *VU1, *S. *Typhi or *S. *Paratyphi A (Table 2). The addition of the internal control did not hinder detection of the target sequences from either *S. *Typhi or *S. *Paratyphi A over a range of DNA concentrations (Table 2).

**Table 2:** Assessment of the reproducibility of the multiplex real-time PCR assay on diluted plasmid DNA containing cloned target sequences

Amplification target	Result	Target copies				
		
		**5 × 10**^**4**^	**5 × 10**^**3**^	**5 × 10**^**2**^	**5 × 10**^**1**^	**5 × 10**^**0**^
*S. *Typhi without internal control	*Ct *value*	22.76	25.61	27.92	31.22	-
*S. *Typhi with internal control	*Ct *value	22.07	25.56	27.76	31.81	-

Intra-assay variation†	CV (%)	0.45	0.25	0.41	0.67	1.01
Inter-assay variation‡	CV (%)	1.86	1	1.97	3.39	1.19

*S. *Paratyphi A without internal control	*Ct *value	21.27	24.78	27.77	31.16	-
*S. *Paratyphi A with internal control	*Ct *value	21.42	24.66	28.28	31.84	-

Intra-assay variation	CV (%)	0.41	0.7	0.67	0.86	1.34
Inter-assay variation	CV (%)	1.34	1.34	0.86	1.25	1.83

* Mean *Ct *value calculated from 4 individual replicates on 4 separate days, n = 16. †Intra-assay variation was calculated by measuring the co-efficient of variance of *Ct *value on 4 concurrently run assays. ‡ Inter-assay variation was calculated by comparing variation in *Ct *value on experiments on 4 individual occasions.

Using serially diluted quantities of plasmid DNA containing *S. *Typhi and *S. *Paratyphi A (extracted from strains VU2 and VU3) target sequences, we assessed the detection limit, reproducibility and quantitative ability of the assay. Table 2 shows the results of consecutive standard curve experiments and demonstrates the overall performance, intra-assay variation and the inter-assay variation. The inter-assay co-efficient of variance ranged from 0.86 to 3.39% with copy number ranging from 5 × 10^1 ^to 5 × 10^5 ^copies per reaction. Repeat standard curve experiments were performed on DNA extracted from PBS and whole blood spiked with *S. *Typhi (Table 3). There was an insignificant variation (*p *> 0.1 with none-parametric student's t-test) in *Ct *value when the PCR assay was performed on DNA extracted from whole blood or PBS inoculated with a known quantity of bacterial cells. The detection limit of the assay ranged from between 1 to 5 target copies per reaction. Therefore, in spiked samples, the real-time PCR method, was specific, sensitive and not influenced by potential inhibitors in blood or by the addition of the *E. coli *internal control.

**Table 3:** Detection limit and *Ct *value comparison of PCR amplification on nucleic acid extractions from inoculated blood, inoculated PBS and purified plasmid DNA

	Amplification targets and *Ct *value					
	
	*S*. Typhi			*S*. Paratyphi A		
Equivalent cfu/ml	PBS	Blood	Plasmid	PBS	Blood	Plasmid
1 × 10^3^	*Ct *value	21.27	24.78	27.77	31.16	-
1 × 10^4^	*Ct *value	21.42	24.66	28.28	31.84	-
1 × 10^4^	CV (%)	0.41	0.7	0.67	0.86	1.34
1 × 10^6^	CV (%)	1.34	1.34	0.86	1.25	1.83

### Performance of PCR assay on biological specimens

We performed the multiplex PCR assay on blood samples taken from 100 culture confirmed enteric fever patients. Fifty four of the 100 blood samples were culture positive for *S. *Typhi and 46 blood samples were culture positive for *S. *Paratyphi A. Both the *S. *Typhi and the *S. *Paratyphi A clinical isolates from these blood samples were verified for the real-time PCR target and all the *S. *Typhi and the *S. *Paratyphi A strains isolated from the corresponding blood samples had the appropriate DNA targets.

**Figure 1:**
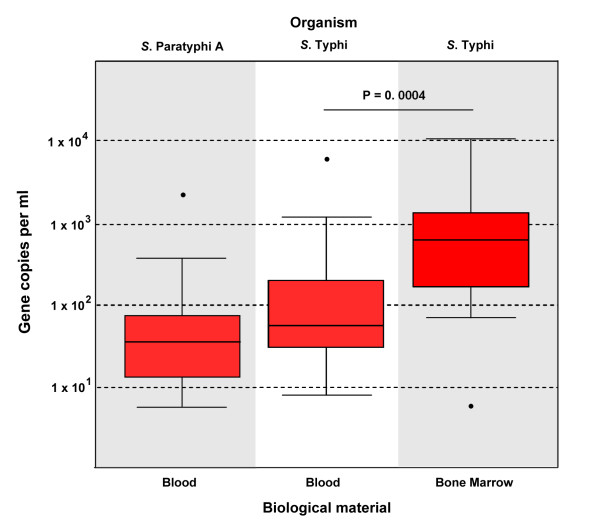
**Real-time PCR amplification of *S*. Typhi and *S*. Paratyphi A in blood and bone marrow specimens from patients with culture confirmed enteric fever**. The *Ct *values of amplification positive blood and bone marrow specimens have been converted by dilution factor to copies per ml of biological material (y axis). The median and quartile ranges of the number of copies per ml of biological sample are shown for amplification positive blood samples with *S*. Paratyphi A (n = 18), amplification positive blood samples with *S*. Typhi (n = 23) and amplification positive bone marrow samples with *S. *Typhi (n = 28). Statistical significance was calculated using a none-parametric student's t-test.

The real-time PCR was performed on DNA extracted from blood taken for microbiological culture at the time of clinical diagnosis, prior to the administration of antimicrobials. All samples were inoculated with *E. coli *VU1 before DNA extraction to ensure reliable DNA isolation and amplification. PCR was performed using 5 μl of DNA taken from a 300 μl re-suspension volume, which correlated with a 4 ml of whole blood; we calculated that the final PCR amplification was performed on an equivalent volume of 75 μl of whole blood. Data resulting from the positive amplicons is shown in Figure 1.

Reliable amplification was obtained from the *E. coli *VU1 internal control strain in all 100 tested samples and no samples produced an amplicon which indicated co-infection with both *S. *Typhi and *S. *Paratyphi A. Serovar specific amplification for *S. *Typhi and for *S. *Paratyphi A was observed in 23 and 18 samples respectively. The multiplex real-time PCR assay, consequently, had a sensitivity of 42% (23/54) for *S. *Typhi and 39% (18/46) for *S. *Paratyphi A. We were unable to amplify *S. *Typhi or *S. *Paratyphi A target DNA from any of the 50 blood samples from enteric fever patients that were culture negative, or from DNA extracted from blood taken from 25 patients with other known causes of bacteraemia; specificity 100% (75/75).

The assay was also performed on DNA extracted from 28 bone marrow biopsies which had been cultured and were known to be positive for *S. *Typhi. Specific amplification of the *S. *Typhi target sequence was detected in DNA extracted from all 28 biopsies, thus giving a sensitivity of 100% (28/28) in these specimens (Figure 1).

Quantitative assessment of the resulting *Ct *values showed that the number of copies of target DNA was lowest for *S. *Paratyphi A, ranging from 5 to 2,000 with a median of 39 copies per ml of blood (Figure 1). The *S. *Typhi positive amplifications ranged from 8 to 6,000 copies per ml, with a median of 60 copies per ml of whole blood. There was a statistically significant increase (none-parametric student's t-test) in target copies per ml in bone marrow samples when compared to blood samples (Figure 1). The number of *S. *Typhi target sequence in bone marrow ranged from 6 to 10,000 with a median of 633 copies per ml.

### Real-time PCR detection limit

We demonstrated that an equivalent *Ct *value could be generated on DNA extracted from bacteria in PBS and whole blood, thus inhibition was not the limiting factor in poor sensitivity on blood specimens. Our data suggested that the lack of positive PCR amplification was due to the low number of organisms in the blood sample, which were below the detection limit of the PCR assay. We compared blood culturing and PCR under experimental conditions. A known quantity of colony forming units of *S. *Typhi were inoculated into whole blood. The sample was equilibrated and 10 fold serial dilutions were performed in whole blood. The diluted blood samples were cultured in order to enumerate organisms, inoculated into BACTEC bottles and incubated. Additionally, total DNA was extracted from all samples and real-time PCR was performed as before. To assess the effect of antimicrobials (PCR may detect dead organisms) we also performed an matching experiment, yet the inoculated blood samples were exposed to gentamicin or ciprofloxacin for 2 hours. Results are presented in Table 4.

**Table 4:** Comparison of real-time PCR detection to blood culture with known inoculants of *S. *Typhi into blood samples

	Experimental condition and detection method								
	No antimicrobial			Gentamicin			Ciprofloxacin		
	
Dilution factor	cfu/ml	*Ct *value	Blood culture	cfu/ml	*Ct *value	Blood culture	cfu/ml	*Ct *value	Blood culture
10^-4^	2.5 × 10^4^	30.18	+	1 × 10^4^	30.44	+	3 × 10^3^	30.31	+
10^-5^	2.5 × 10^3^	33.53	+	1 × 10^3^	33.45	+	3 × 10^2^	33.44	+
10^-6^	2.5 × 10^2^	37.77	+	1 × 10^2^	37.07	+	3 × 10^1^	36.95	+
10^-7^	2.5 × 10^1^	-	+	1 × 10^1^	-	+	3 × 10^0^	-	+
10^-8^	<2.5 × 10^1^	-	+	<1 × 10^1^	-	+	<3 × 10^3^	-	-

Target DNA was consistently amplified in samples up to the sixth 10 fold dilution, which corresponded to 2.5 × 10^2 ^DNA copies per ml of blood (Table 4). Amplification was not prevented when samples were treated with antimicrobials. Culturing of the inoculated blood samples in BACTEC bottles was consistently more sensitive than PCR amplification, both in the presence or absence of antimicrobials (Table 4).

## Discussion

A molecular method for the detection of invasive *Salmonella *serovars in biological specimens appears to be an attractive addition to current methods. However, PCR is not commonly reported for the routine identification of invasive *Salmonellae*, this is in spite of a number of publications demonstrating its potential use in the clinical setting for diagnostic testing and bacterial identification [12,21-23]. It is assumed that PCR amplification may be a suitable test where blood culturing is not routinely performed. A potential advantage of PCR is that if it had a high level of sensitivity it may be performed on smaller volumes of blood than required for culture and may have the added ability of detecting dead organisms. Such permutations may be probable in an endemic setting when taking blood from young children and when patients have access to none-prescribed antimicrobials.

Many of the previously published *S. *Typhi nucleic acid detection studies harbour limitations; the methodology is often inappropriately validated, equivalent blood volumes are not specified, the primers are nested and target the flagellin (*fliC*) gene and detection is via conventional agarose gel electrophoresis [13,15]. All these limitations may cause results with may not be reproducible and hinder the accurate amplification of target sequences in biological samples. Furthermore, many such reports suggest the usefulness of the technique in patients where enteric fever cannot be confirmed by other methods. Whilst the rapid nature of a PCR assay may compensate for many potential limitations, a balanced assessment of PCR sensitivity in a clinical setting was required. Real-time PCR addresses many of the limitations that can occur with conventional PCR. The system is sensitive, stringent and less prone to contamination with DNA from other organisms.

The data presented here may also have some limitations, including, the volume of nucleic acid used in the experimental procedure, the blood samples originating from one location and a period of storage prior to DNA extraction. Nonetheless, this work represents an unbiased assessment of PCR in the identification of *S. *Typhi and *S. *Paratyphi A in biological specimens. The blood inoculation and bacterial quantification experiments support our findings on biological specimens and address some of the limitations from biological samples.

We attribute a lack of sensitivity of the assay to the low physiological level of invasive *Salmonella *organisms in the blood. The detection limits of the real-time PCR were comparable with cfu/ml in both inoculated blood and saline samples, demonstrating that human DNA or potential PCR inhibitors found in blood may not hinder amplification. We additionally found that a realistic detection limit of the assay was between 100 to 200 organisms per ml of whole blood. This may be increased by extracting DNA from a greater blood volume or by precipitation of the extracted DNA. Both improvements would be technically challenging and even if these limitations are taken into account, PCR may still fail to reach the sensitivity level of a standard blood culture.

Our quantitative data demonstrated median copies of target sequence of 39 and 60 per ml of blood for *S. *Paratyphi A and *S. *Typhi respectively and 600 copies of *S. *Typhi target per ml of bone marrow. These data are somewhat incomparable with a previous real-time PCR detection assay for *S. *Typhi in peripheral blood [10]. Massi *et al*. found a statistically significant difference between hypothetical loads of bacteria in blood between culture negative and culture positive blood specimens. Patients that were culture positive had between 1,010 and 4,350 target copies per ml of blood, whereas, patients that were culture negative had between 3.9 and 990 copies per ml of blood. Even taking into account dead organisms, these figures correspond with substantial bacterial loads in the blood of enteric fever patients. This discrepancy is an important observation as it has been shown that *S. *Typhi induces febrile disease with a nominal number of organisms circulating in the blood. Using quantitative counts of bacteria in blood, Wain *et al. *demonstrated that 25% of all acute typhoid patients had less than 0.1 cfu/ml and only 1% tested had a cfu/ml of greater than 100 organisms per ml of blood [24]. Our PCR results concur with these data and supports our understanding that the lack of sensitivity is dependent on the low number of invasive *Salmonellae *in the blood. Therefore, to detect a living organism, the PCR would have to be performed directly on DNA extracted from 10 ml of blood. A lack of detectable organisms is a potential consideration for other bacterial pathogens, such as *Mycobacterium tuberculosis*; meta-analyses suggest that PCR detection of this organism in biological material may also pose a similar challenge [25,26].

It is of note, however, that the PCR assay demonstrated a sensitivity of 100% on culture positive bone marrow biopsies. Bacterial loads in bone marrow biopsies from enteric fever patients have been shown to be significantly higher than bacterial loads in peripheral blood [16]. Additionally, the tenfold increase in copies per ml in bone marrow, when compared to blood, may be explained not only by the organisms surviving within macrophages in the bone marrow, but also the potential ability of the assay to detect DNA from dead organisms within cells. A combination of culturing, either from blood or bone marrow, with PCR amplification may improve sensitivity and time to diagnosis. It is clear that typhoid diagnostics requires the use of some new approaches and fresh considerations [27].

## Conclusions

Our data demonstrates that a low level of bacteria in the blood makes PCR amplification of specific *S. *Typhi and *S. *Paratyphi A sequences on biological samples technically challenging. Whilst specificity for the technique is indisputably high, the sensitivity when compared to blood culturing is low. Further assessment of the use of PCR amplification for the detection of invasive *Salmonellae *in blood is required. Previous publications have demonstrated that PCR is both a specific and highly sensitive method for detection of *S*. Typhi in blood. Our study questions the use of PCR for the diagnosis of enteric fever and suggests that the number of organisms and the volume of blood required for accurate identification using PCR on biological samples may be un-physiological and impractical.

## Competing interests

The authors declare that they have no competing interests.

## Authors' contributions

TVTN, AK, SD1, HNT, LTPT performed the experiments. SK, SD2, AA, BB and CD provided biological material and experimental input. KH performed the bioinformatic analysis. TTC and JIC cultured the micro-organisms used. NVVC, JF and SB conceived the study and prepared the manuscript. All authors have read and approved the final manuscript.

## Pre-publication history

The pre-publication history for this paper can be accessed here:

http://www.biomedcentral.com/1471-2334/10/125/prepub
